# MiR-519d impedes cisplatin-resistance in breast cancer stem cells by down-regulating the expression of MCL-1

**DOI:** 10.18632/oncotarget.15781

**Published:** 2017-02-28

**Authors:** Qing Xie, Shuai Wang, Yue Zhao, Zhenchao Zhang, Chuan Qin, Xianjun Yang

**Affiliations:** ^1^ Tumor Signaling and Transduction Lab, School of Basic Medical Sciences, Xinxiang Medical University, Xinxiang City, Henan Province, 453003, PR China; ^2^ Department of Human Parasitology, School of Basic Medical Sciences, Xinxiang Medical University, Xinxiang City, Henan Province, 453003, PR China

**Keywords:** BCSCs, miR-519d, cisplatin, resistance, MCL-1

## Abstract

Cancer stem cells are considered as the cell population which is responsible for chemoresistance and treatment failure in breast cancer patients. Therefore, it is urgent to explore the mechanism by which cancer stem cells survive under the treatment of chemotherapeutic drugs such as cisplatin. In this paper, we demonstrated significant decrease of miR-519d in breast cancer stem cells by quantitative RT-PCR analysis. Furthermore, we found the enforced expression of miR-519d in T-47D-cancer stem cells significantly increased their sensitivity to cisplatin through the apoptosis pathway. In addition, the gene of MCL-1, which is a member of pro-apoptotic Bcl-2 family, was found to be the target of miR-519d in T-47D-cancer stem cells. Our date demonstrated that enforced miR-519d expression enhanced the cisplatin-induced apoptosis through the MCL-1 dependent mitochondria pathway in breast cancer stem cells. Taken together, the present study suggests that miR-519d reduces chemoresistance in breast cancer stem cells, and understanding of miR-519d may be helpful for increasing the efficacy of chemotherapy.

## INTRODUCTION

Breast cancer (BC) is reported to be the common cancer, which accounts for leading cause of cancer-related death worldwide among women [1]. Breast cancer stem cells (BCSCs) are recognized as a small group of highly tumorigenic ability cells in tumor, which are endowed with self-renewal and associated with the chemoresistance, recurrence, and metastasis [2, 3]. Previous studies have identified the CD44^+^/CD24^−/low^ phenotype as the surface marks of BCSCs, which were firstly isolated by Al-Hajj and shown high tumorigenicity in immunocompromised mice [4]. In addition to the high tumorigenicity, CSCs are considered as the group of cells which are responsible for chemoresistance and treatment failure in BC patients [5]. Therefore, it is urgent to explore the mechanism by which BCSCs survive under the treatment of chemotherapeutic drugs.

Cisplatin is a high efficient-spectrum anticancer drug, which is widely used to treat multiple malignant tumors, such lung cancer, head and neck cancer cancer, gastric cancer, and breast cancer [6–9]. It induces the apoptosis of tumor cells by crosslinking with the DNAs to block DNA replication and transcription [10]. Unfortunately, repetitive and long-term administration of cisplatin usually induces severe drug-resistance in cisplatin treated breast cancer cells [11]. It's urgent to improve the sensitivity of BC cells to cisplatin. Since the recent researches have suggested that the cisplatin-resistance is associated with the cancer stem cells, we then investigated the role of miR-519d in cisplatin-resistance in BCSCs.

MicroRNAs (miRNAs) are a class of non-coding RNAs, which are endogenous and involved in post-transcriptional regulation of about 60% of the human genes by binding to target mRNAs at the 3′untranslated region (3′ UTR) [12, 13]. They are involved in post-transcriptional control of approximately 60% of the human genes by binding to the 3′-untranslated region (3′-UTR) of target mRNAs. Therefore, the normal expression profile of miRNAs is required in various processes including cell proliferation, differentiation, metabolism and apoptosis. Dysregulation of miRNAs is associated with multiple human diseases including cancer [14, 15]. Previous studies have identified that miR-519d acts as a tumor suppressor in several cancers. For instance, miR-519d could suppress the cell growth of hepatocellular carcinoma cells by inhibiting the MKi67 gene [16]. In breast cancer, studies demonstrated that the miR-519d-mediated downregulation of STAT3 inhibited the cell proliferation and invasion of tumor cells [17]. Apart from these, the miR-519d was also reported to enhance the cisplatin-mediated cytotoxicity to the ovarian cancer cells [18]. However, the role of miR-519d in the cancer stem cells (CSCs) remains unclear. In this study, we found that miR-519d was decreased in breast cancer. Since the recent researches have suggested that the cisplatin-resistance is associated with the cancer stem cells [19], we then investigated the role of miR-519d in cisplatin-resistance in BCSCs, which might serve as a potential strategy for BC therapy.

## RESULTS

### MiR-519d is decreased in breast cancer stem cells

**Figure 1:**
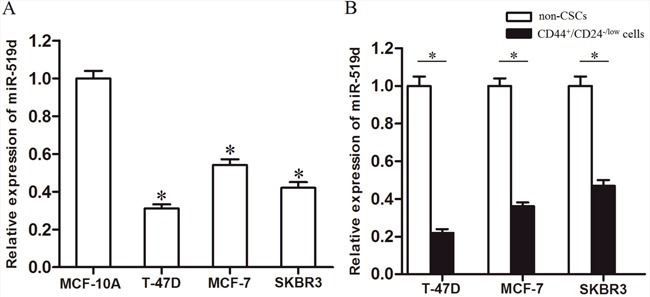
MiR-519d is down-regulated in breast cancer stem cells **A**. The relative expression levels of miR-519d were detected by qRT-PCR in MCF-10A, T-47D, MCF-7 and SKBR3 cells. *P<0.05 *vs*. MCF-10A cells. **B**. The breast cancer stem cell (BCSC) population was sorted as CD44^+^/CD24^−/low^ cells. The expression levels of miR-519d in T-47D, MCF-7 and SKBR3 BCSCs and the corresponding non-CSCs were detected by qRT-PCR analysis, *P<0.05.

To investigate the potential role of miR-519d in breast cancer, we compared with the expression levels of miR-519d between three BC cell lines and the MCF-10A which is the non-tumorigenic epithelial cell line. As shown in Figure 1A, miR-519d was found to be decreased in T-47D, MCF-7 and SKBR3 BC cells lines compared with that in MCFL-10A. Interestingly, the analyses of expression levels of miR-519d in BC cell lines demonstrated that the levels of miR-519d in T-47D-CSCs, MCF-7-CSCs, and SKBR3-CSCs are significantly lower than those in their corresponding non-CSCs (Figure 1B). These results suggested that miR-519d may play important roles in breast cancer stem cells.

### MiR-519d sensitizes the BCSCs to cisplatin treatment

**Figure 2:**
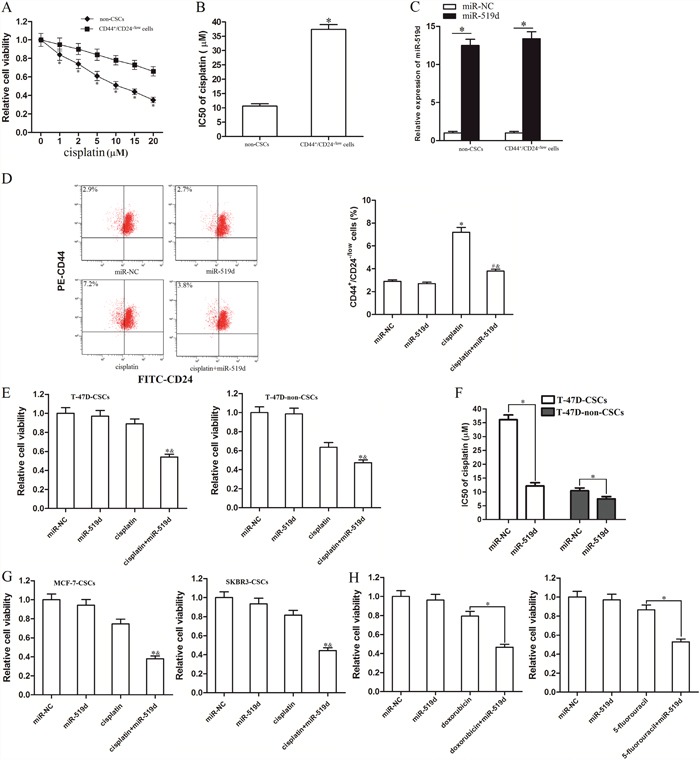
MiR-519d sensitizes the T-47D-CSCs to cisplatin treatment **A**. The sorted T-47D-CSCs and non-CSCs were treated with different concentrations of cisplatin. 48 h later, the MTT assay was performed to measure the cell viability. *P<0.05 *vs*. the corresponding non-CSCs treated with the equal concentration of cisplatin. **B**. The IC50 of cisplatin to T-47D-CSCs and the non-CSCs was calculated according to the cell viability curves performed by MTT assay. *P<0.05 *vs*. non-CSCs. **C**. The sorted T-47D-CSCs and non-CSCs were transfected with 50 pmol/ml miR-NC or miR-519d. 24 h later, the transfection efficiency was measured by qRT-PCR analysis. *P<0.05. **D**. The effect of miR-519d and cisplatin (5 μM) on changing the percentage of the CSC population of T-47D was evaluated by flow cytometry using CD44 and CD24 antibodies. *P<0.05 *vs*. miR-NC group. ^#^*P*<0.05 *vs*. cisplatin group. ^&^*P*<0.05 *vs*. miR-519d group. **E**. Effect of miR-519d on cisplatin-induced (5 μM) cell death in T-47D-CSCs and T-47D-non-CSCs.*P<0.05 *vs*. cisplatin group. ^&^*P*<0.05 *vs*. miR-519d group. **F**. IC50 of cisplatin to T-47D-CSCs and T-47D-non-CSCs. *P<0.05. **G**. Effect of miR-519d on cisplatin-induced (5 μM) cell death in MCF-7-CSCs and SKBR3-CSCs. *P<0.05 *vs*. cisplatin group. ^&^*P*<0.05 *vs*. miR-519d group. **H**. After treatment with miR-519d, doxorubicin (0.5 μg/ml) and 5-fluorouracil (1 μM), cell viability of T-47D-CSCs was detected by MTT assay. *P<0.05.

To investigate the sensitivity of BCSCs and non-CSCs to cisplatin treatment, we sorted the T-47D-CSCs and their corresponding non-CSCs using the surface marks of CD44 and CD24. According to the results of MTT assays, we confirmed that the sensitivity of T-47D-CSCs to cisplatin was significantly lower than the non-CSCs (Figure 2A). Intuitively, the IC50 of cisplatin to T-47D-CSCs was 2.5-fold higher than the non-CSCs (Figure 2B). To explore the role of miR-519d in T-47D-CSCs, the miR-519d mimic was transfected into the T-47D-CSCs and non-CSCs (transfection efficiency is shown Figure 2C). In addition, we chose 5 μM cisplatin for combination treatment with miR-519d mimics, because this concentration of cisplatin induced slight cell death in T-47D-CSCs (Figure 2A). Interestingly, treatment with cisplatin in T-47D increased the percentage of the CSC population defined as CD44^+^CD24^−/low^ cells. However, combination with miR-519d mimic significantly inhibited the effect of cisplatin on enriching the CSC population (Figure 2D). We explain that the treatment of cisplatin alone probably killed the cisplatin-sensitive non-CSCs and survival of cisplatin-resistant T-47D-CSCs. Moreover, transfection of miR-519d may increase the sensitivity of T-47D-CSCs to cisplatin. Results of MTT assays showed that miR-519d strongly promoted the cisplatin-induced cell death in T-47D-CSCs, whereas the effect of miR-519d was slight on enhancing the anti-tumor effect of cisplatin on T-47D-non-CSCs (Figure 2E). Intuitively, the IC50 of cisplatin to miR-519d transfected T-47D-CSCs was 69.0% lower than the miR-NC transfected T-47D-CSCs. In contrast, miR-519d reduced the IC50 of cisplatin to T-47D-non-CSCs only by 28.1% (Figure 2F). We therefore demonstrated that T-47D-CSCs were more sensitive to miR-519d-promoted cell death rather than their corresponding T-47D-non-CSCs. Moreover, we found that miR-519d also promoted the cisplatin-induced cell death in other kinds of BCSCs such as MCF-7-CSCs and SKBR3-CSCs (Figure 2G). It's proved that miR-519d sensitized the BCSCs to cisplatin treatment. In addition, After combination treatment with miR-519d mimic and doxorubicin or 5-fluorouracil in T-47D-CSCs, we observed that transfection with miR-519d also enhanced the anti-tumor effect of them (Figure 2H). These data suggested that miR-519d may increase the chemosensitivity of BCSCs.

### MiR-519d enhances the anti-tumor effect of cisplatin *in vivo*

**Figure 3:**
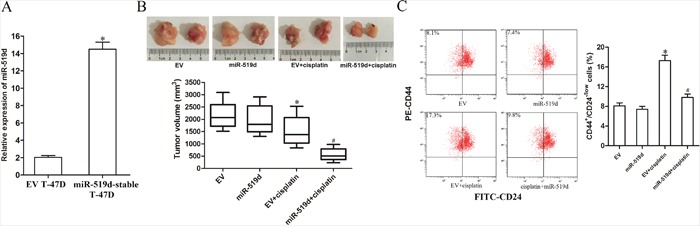
MiR-519d enhances the anti-tumor effect of cisplatin *in vivo* **A**. Expression levels of miR-519d in miR-519d-stable T-47D cells and the corresponding routine T-47D cells (EV T-47D) were detected by qRT-PCR analysis, *P<0.05 vs. EV T-47D. **B**. Volumes of T-47D tumor tissues were measured after the mice were euthanized on 31 days post-injection. *P<0.05 vs. EV group. ^#^*P*<0.05 vs. EV+cisplatin group. **C**. The population of CSCs in T-47D tumor tissues was measured by flow cytometry. *P<0.05 vs. EV group. ^#^*P*<0.05 vs. EV+cisplatin group.

To explore the effect of miR-519d on cisplatin therapy *in vivo*, T-47D cells with stable-overexpressed miR-519d were inoculated to the mice. The expression profile of miR-519d in miR-519d-stable T-47D and routine T-47D was shown in Figure 3A. We found that the average volume of tumors in the group of miR-519d was significantly smaller than the group of EV when the two groups of mice were treated with equal dose of cisplatin (Figure 3B). It demonstrated that overexpression of miR-519d enhances the anti-tumor effect of cisplatin *in vivo*. Additionally, we observed that cisplatin single treatment significantly increased the percentage of CSCs population in T-47D tumor tissues. However, overexpression of miR-519d abolished the enrichment of CSCs population induced by cisplatin (Figure 3C). These results indicated that miR-519d increases the sensitivity of breast cancer cells to cisplatin by targeting CSCs *in vivo*.

### MiR-519d targets the MCL-1 in the BCSCs

**Figure 4:**
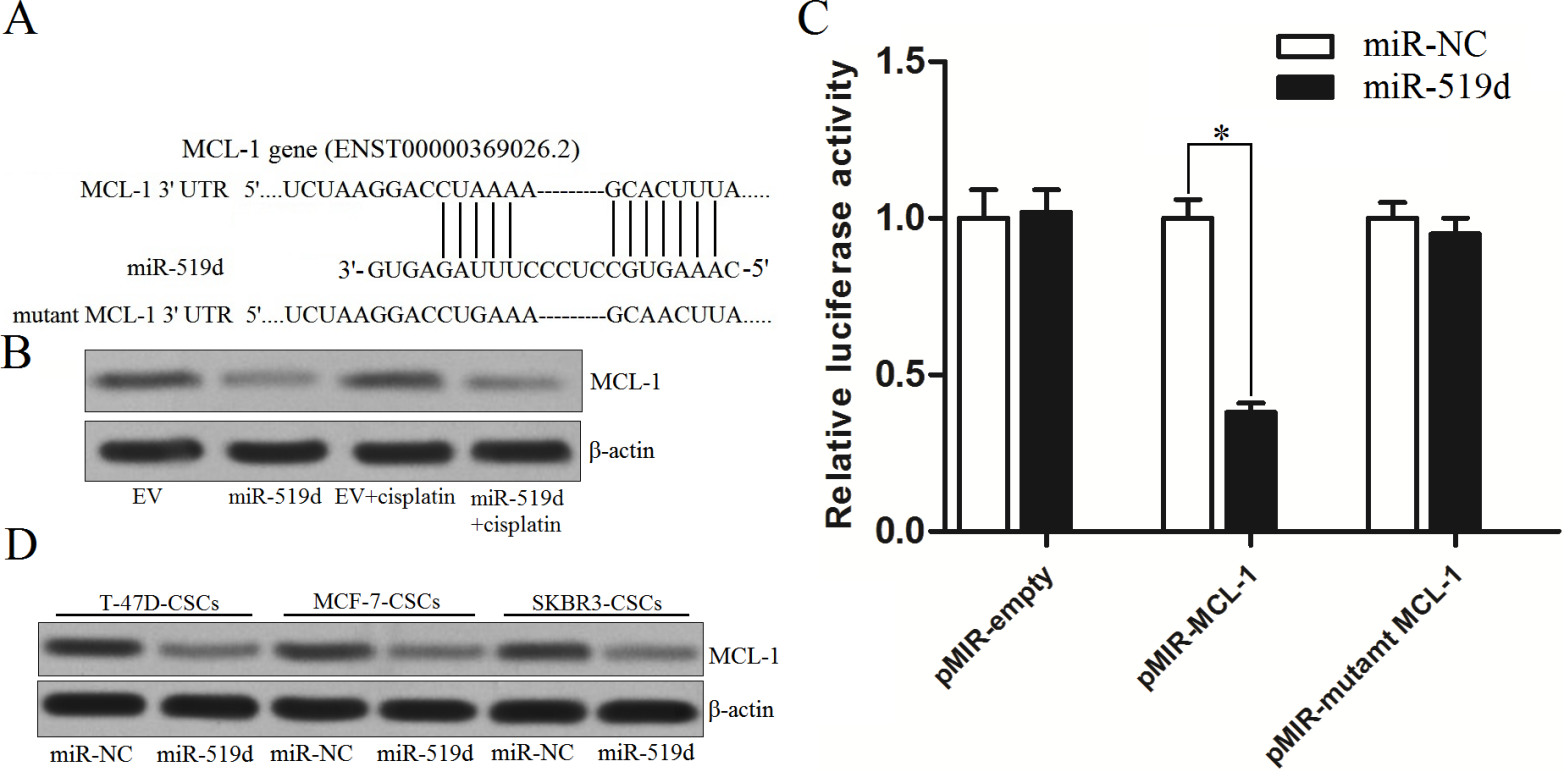
MCL-1 is the target of miR-519d **A**. MCL-1 mRNA 3′ UTR was predicted as the target of miR-519d predicted by the TargetScan, miRanda, and PicTar databases. **B**. Western blot analysis was performed to detect the expression of MCL-1 in T-47D tumor tissues. **C**. T-47D-CSCs were co-transfected with the wildtype/mutant 3′-UTR of pMIR-MCL-1 plasmid and the miR-519d mimics. 48 h post transfection, the luciferase reporter assay was performed using the Dual Luciferase Assay System according to the manufacturer's instruction. *P<0.05. **D**. Expression level of MCL-1 in the T-47D, MCF-7 and SKBR3 BCSCs after they were transfected with miR-519d.

To understand the mechanism by which miR-519d facilitates cisplatin-induced cell death, the targets of miR-519d were predicted by three miRNA databases (TargetScan, miRanda, and PicTar). Among the potentially targeted genes, it was revealed that the gene of MCL-1 was commonly predicted by all of these public databases (Figure 4A). As the MCL-1 is an important anti-apoptotic protein in cancers [20], we speculated that the miR-519d may promote the cisplatin-induced cell death by down-regulating the expression of MCL-1 in the T-47D-CSCs. To investigate this hypothesis, we detected the expression of MCL-1 in T-47D tumor tissues. We found that the cells transfected with lentivirus carrying miR-519d expressed significantly lower level of MCL-1 compared with that in the EV group (Figure 4B). Next, we performed luciferase reporter assays in the T-47D-CSCs *in vitro*. As shown in Figure 4C, transfection with miR-519d significantly decreased the luciferase activities of pMIR-MCL-1 plasmid but not the pMIR-mutant MCL-1 plasmid or the empty one. Furthermore, the results of western blot analysis demonstrated that the miR-519d actually decreased the expression level of MCL-1 in the T-47D, MCF-7 and SKBR3 BCSCs (Figure 4D). Taken together, these results indicated that the MCL-1 gene is a functional target of miR-519d in the T-47D-CSCs.

### MiR-519d sensitizes T-47D-CSCs to cisplatin-induced cell death through the miR-519d/MCL-1 axis

**Figure 5:**
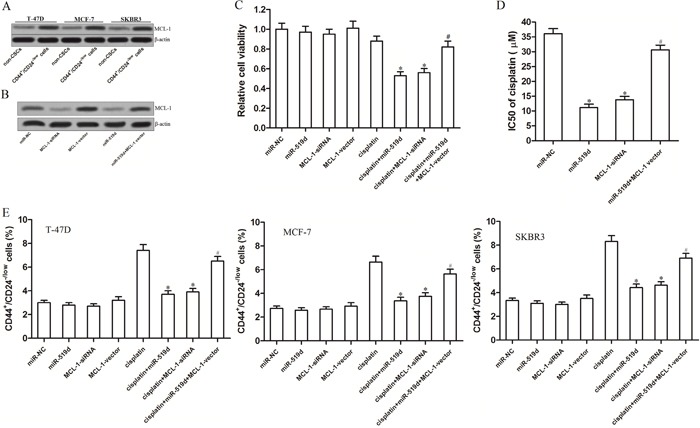
MiR-519d sensitizes T-47D-CSCs to cisplatin-induced cell death through the miR-519d/MCL-1 axis **A**. Western blot analysis of the expression of MCL-1 in the T-47D, MCF-7 and BKBR3 BCSCs and the corresponding non-CSCs. **B**. The transfection efficiency of MCL-1 siRNA and MCL-1 vector in the T-47D-CSCs. **C**. The cell viability of T-47D-CSCs treated with cisplatin (5 μM), RNAs and plasmid. *P<0.05 *vs*. cisplatin group, ^#^*P*<0.05 *vs*. cisplatin+miR-519d group. **D**. The IC50 of cisplatin in the T-47D-CSCs transfected with RNAs and plasmid. *P<0.05 *vs*. miR-NC group, ^#^*P*<0.05 *vs*. miR-519d group. **E**. The percentage of the CSC population of T-47D, MCF-7 and SKBR3 cells treated with cisplatin (5 μM), RNAs and plasmid. *P<0.05 *vs*. cisplatin group, ^#^*P*<0.05 *vs*. cisplatin+miR-519d group.

As the expression of miR-519d was decreased in the BCSCs (Figure 1B), we observed that the MCL-1 protein was overexpressed in the T-47D, MCF-7 and SKBR3 BCSCs compared with the non-CSCs as expectedly (Figure 5A). It is suggested that absence of miR-519d induced overexpression of MCL-1 in BCSCs, and the miR-519d/MCL-1 axis may play a key role in the sensitivity of BCSCs to the cisplatin-induced cell death. Therefore, we changed the expression of MCL-1 in the T-47D-CSCs by transfecting with the MCL-1 siRNA and the MCL-1 eukaryotic expression vector. The effect of the MCL-1 siRNA and the MCL-1 vector was shown in Figure 5B. Next, we performed the cell viability assay to test the role of MCL-1 in the miR-519d-promoted cell death induced by the cisplatin. We found that knockdown of MCL-1 mediated by the RNA interference also increased the sensitivity of T-47D-CSCs to cisplatin. On the contrary, enforced expression of MCL-1 impaired the promotion of miR-519d to the cisplatin-induced cell death (Figure 5C). Intuitively, the IC50 of cisplatin to miR-519d transfected T-47D-CSCs was 69.0% lower than the miR-NC transfected T-47D-CSCs; the IC50 of cisplatin to MCL-1 siRNA transfected T-47D-CSCs was 61.8% lower than the miR-NC transfected T-47D-CSCs; the IC50 of cisplatin to miR-519d and MCL-1 vector co-transfected T-47D-CSCs was only 15.2% lower than the miR-NC transfected T-47D-CSCs (Figure 5D). In addition, both the miR-519d and MCL-1 siRNA inhibited the enrichment of T-47D, MCF-7 and SKBR3-CSCs population induced by cisplatin, whereas the transfection of MCL-1 vector abolished the effect of miR-519d (Figure 5E). Taken together, these results demonstrated that miR-519d sensitizes the BCSCs to cisplatin-induced cell death through the miR-519d/MCL-1 axis.

### Combination with miR-519d and cisplatin induces caspases-dependent apoptosis in T-47D-CSCs

**Figure 6:**
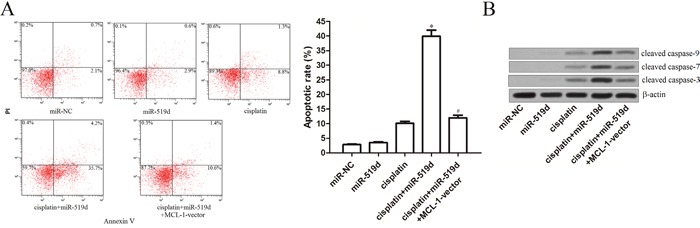
Combination with miR-519d and cisplatin induces caspases-dependent apoptosis in T-47D-CSCs **A**. Flow cytometry analysis was performed to detect the apoptosis in the T-47D-CSCs treated with the miR-519d, cisplatin (5 μM), and MCL-1 vector. *P<0.05 *vs*. cisplatin group, ^#^*P*<0.05 *vs*. cisplatin+miR-519d group. **B**. After treatment with miR-519d, cisplatin (5 μM), and MCL-1 vector, western blot analysis was performed to detect the activation of caspase-9, -7 and -3 in the T-47D-CSCs.

Since the gene of MCL-1 which is the target of miR-519d functions as a anti-apoptotic protein [17], we next investigated whether the miR-519d promoted the cisplatin-induced cell death through the apoptosis pathway in T-47D-CSCs. We observed that although the miR-519d didn't induce significant apoptosis in the T-47D-CSCs, it dramatically enhanced the cisplatin-dependent apoptosis (Figure 6A). Furthermore, the results of western blot analysis demonstrated that the caspase-9, -7 and -3 were significantly triggered due to the combination with the miR-519d and cisplatin in the T-47D-CSCs (Figure 6B). In addition, transfection with MCL-1 vector inhibited both the apoptosis and the caspases activation in the miR-519d plus cisplatin-treated T-47D-CSCs (Figure 6A and 6B). Taken together, these results indicate that the combination with miR-519d and cisplatin induces the caspases-dependent apoptosis in the T-47D-CSCs.

### Combination with miR-519d and cisplatin induces apoptosis through the mitochondria pathway in T-47D-CSCs

**Figure 7:**
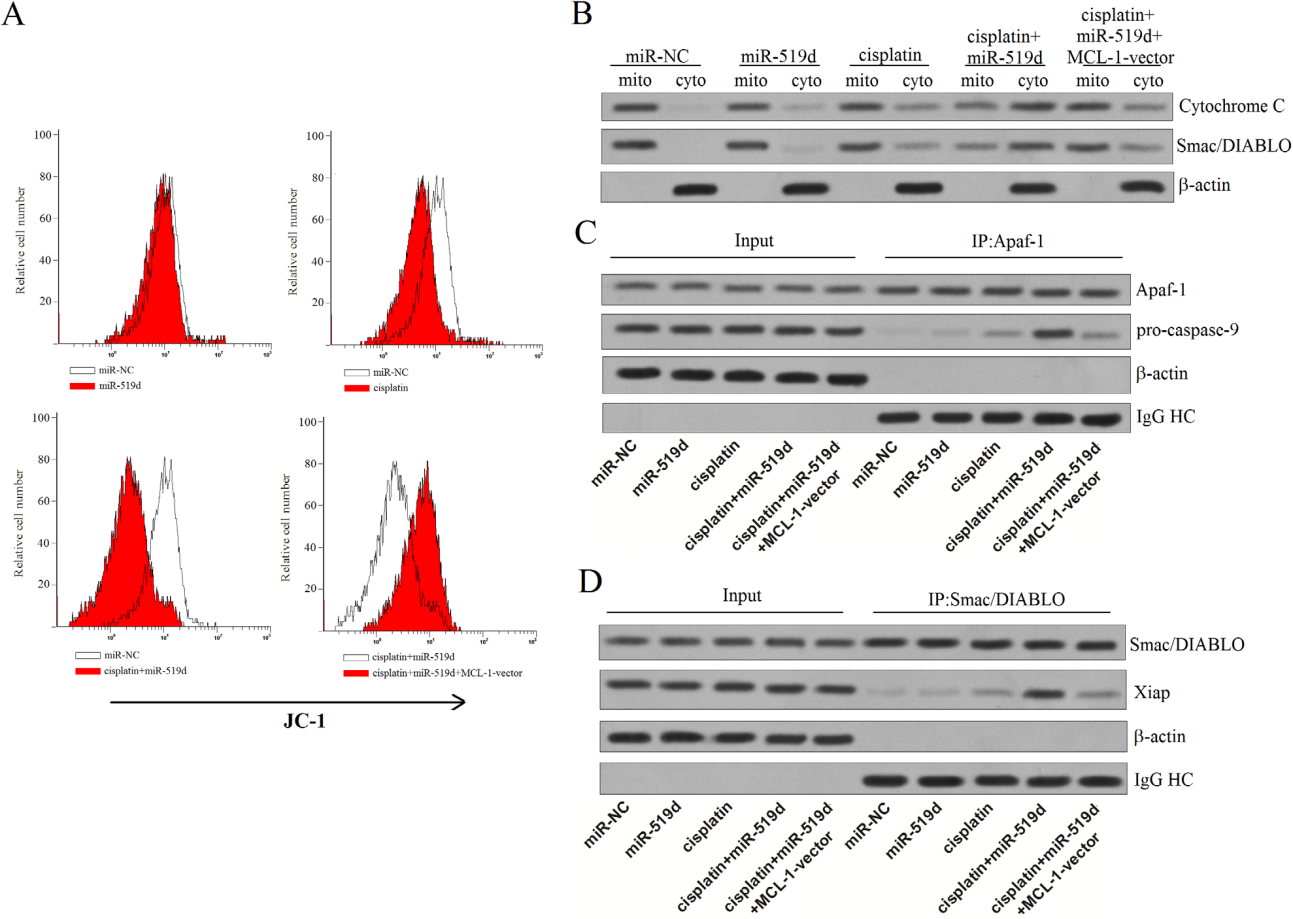
Combination with miR-519d and cisplatin induces apoptosis through the mitochondria pathway in T-47D-CSCs **A**. Flow cytometry analysis was performed to detect the MMP of T-47D-CSCs treated with the miR-519d, cisplatin, and MCL-1 vector. **B**. After the mitochondria were isolated, the protein levels of cytochrome C and Smac/DIABLO in the cytoplasm and mitochondria were measured by western blot analysis. **C**. Co-immunoprecipitation and western blot analysis was performed to detect the formation of Apaf-1/caspase-9 complex. **D**. Co-immunoprecipitation and western blot analysis was performed to evaluate the inaction with the Smac/DIABLO and Xiap.

As the MCL-1 has been proved to inhibit the dysfunction of mitochondria, we investigated the effect of miR-519d on the mitochondria pathway. We observed the cisplatin-dependent mitochondria dysfunction was significantly enhanced when the miR-519d was introduced. As expected, the MCL-1 vector impaired the effect of miR-519d (Figure 7A). We next isolated the mitochondria from the T-47D-CSCs treated with miR-519d and cisplatin. Interestingly, we found the cytochrome C and the Smac/DIABLO, both of which are the mitochondria-derived apoptogenic proteins, were significantly released into the cytoplasm when the miR-519d was transfected into the cisplatin-treated T-47D-CSCs. In contrast, the single treatment with the miR-519d or cisplatin induced the release of cytochrome C and the Smac/DIABLO only slightly (Figure 7B). As the results, the combination with the miR-519d and cisplatin induced the formation of Apaf-1/ caspase-9 complex in the presence of cytochrom C [21] (Figure 7C). Meanwhile, the caspases inhibitor Xiap was also inactivated in the presence of Smac/DIABLO [22] (Figure 7D). In summary, these results suggest that combination with miR-519d and cisplatin can induce the apoptosis of T-47D-CSCs through the mitochondria pathway.

## DISCUSSION

Accumulating evidence indicate that the CSCs are resistant to the chemotherapy, which should be responsible for the low curative effect and tumor recurrence [23, 24]. Therefore, it's significant to increase the chemosensitivity of CSCs. In the present study, we performed several tests to investigate the role of miR-519d in changing the sensitivity of cisplatin to the T-47D-CSCs. We then discovered that the transfection of miR-519d could enhance the cytotoxicity of cisplatin and decrease the IC50 of it to the T-47D-CSCs. Interestingly, the cisplatin-treatment increased the percentage of the CSC population to the unsorted T-47D cells. We inferred this phenomenon may be caused by the lower sensitivity of BCSCs compared with the non-CSCs. As the results of the transfection with miR-519d, the cisplatin-dependent increased of CSC population was inhibited obviously. Therefore, we declared that the dysregulation of miR-519d may be an important factor to induce the chmoresistance in BCSCs.

Myeloid cell leukemia 1 (MCL-1) is an anti-apoptotic protein belongs to the Bcl-2 family. As the MCL-1 owns three BH (Bcl-2 homology) domains, it can interact with the pro-apoptotic proteins such as Bax, Bim, Noxa and Puma. As the results of the binding, the MCL-1 inhibits the apoptosis pathway by inactivating these pro-apoptotic proteins [25, 26]. Researches have revealed that the MCL-1 is usually overexpressed and mediates the chemotherapy resistance in multiple cancers. Therefore, the MCL-1 could be considered as an important target for the cancer therapy, and knockdown of MCL-1 has been reported to increase the anti-tumor effect of chemotherapeutic drugs including cisplatin [27, 28]. In the present study, we found the overexpression of miR-519d directly decreased the expression of MCL-1 in the T-47D-CSCs. Furthermore, we proved the miR-519d/MCL-1 axis controlled the sensitivity of T-47D-CSCs to the cisplatin-treatment.

Since the MCL-1 locates on the mitochondrial membrane and inhibits the mitochondria apoptosis [29], we focused on the function of mitochondria to study the apoptosis pathway activated by the combination with the miR-519d and cisplatin. According to the results of the flow cytometry analysis, we demonstrated that combination with miR-519d and cisplatin induced the mitochondria apoptosis in T-47D-CSCs by opening the mitochondrial membrane permeability transition pore and subsequently releasing the cytochrome C and Smac/DIABLO which are the pro-apoptotic inducers from the mitochondria [30, 31]. Previous studies indicated the cytochrome C induced apoptosis by triggering the Apaf-1/caspase-9 apoptosome, and the Smac/DIABLO played its role by neutralizing the X-linked inhibitor of apoptosis protein (Xiap) [32, 33]. Consistent with these researches, we found the cytoplasmic cytochrome C and Smac/DIABLO triggered the formation of the Apaf-1/caspase-9 apoptosome as well as neutralizing the Xiap in the T-47D-CSCs co-treated with miR-519d and cisplatin.

**Figure 8:**
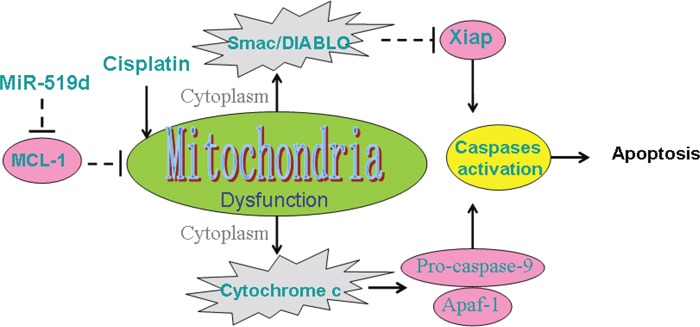
Schema of mitochondrial apoptosis pathway in T-47D-CSCs response to cisplatin and miR-519d Combination with miR-519d and cisplatin induces the damage of mitochondria, resulting in the release of cytochrome C and Smac/DIABLO. Subsequently, caspases are activated and apoptosis is occurred.

In summary, our data demonstrated the key role of miR-519d in promoting the anti-tumor effect of cisplatin on BCSCs through the mitochondria apoptosis pathway (Figure 8). Although the drug-resistance is a major challenge for the cisplatin-treatment to the CSCs [34], the strategy for overexpressing the miR-519d may be a potential approach to increase the sensitivity of BCSCs to cisplatin.

## MATERIALS AND METHODS

### Cell lines

BC cells lines T-47D, MCF-7, SKBR3 and the MCF-10A cell line which is considered as the non-tumorigenic mammary epithelial cells [35] were originated from ATCC. The BC cells were grown in DMEM medium supplemented with 10% fetal calf serum. MCF-10A cells were grown in DMEM/F-12 medium contained 5 % horse serum, 0.5 μg/ml hydrocortisone, 20 ng/ml epidermal growth factor, 100 ng/ml cholera toxin and 10 μg/ml insulin. Cells were grown at 37°C with 5% CO_2_.

### Real-time polymerase chain reaction (RT-PCR) for miR-519d expression

Total RNA from the BC cell lines and MCF-10A were extracted by RNAiso Reagent Plus (Takara, China). Reverse transcription reaction for miR-519d was conducted by using One Step PrimeScript miRNA cDNA Synthesis Kit (TaKaRa), according to the manufacturer's protocols. RT-PCR for miR-519d expression was conducted by using SYBR Premix Ex Taq (TaKaRa) according to the manufacturer's protocols. The relative expression of miR-519d was normalized to U6 snRNA by using the 2^−ΔΔCT^ analysis method [36].

### Flow cytometry analysis and cell sorting

CD24-FITC and CD44-PE antibodies (BD Pharmingen, USA) were used for flow cytometry analysis and cell sorting of BCSCs. Briefly, cells were incubated with the above antibodies on ice for 40 min in the dark. After washing with cold PBS, CD44^+^CD24^−/low^ BCSCs were purified from T-47D, MCF-7 and SKBR3 cell lines.

### Transfection

MiR-519d mimics was synthesized for overexpression of miR-519d. MCL-1 siRNA was synthesized for knockdown of MCL-1 gene. MiR-NC was random RNA oligonucleotides used for negative control. All of these RNAs were purchased from RiboBio Co. Ltd. (China). For enforced expression of MCL-1, MCL-1 open reading frame was amplified to the pEGFP-N1 eukaryotic expression vector (Clontech, USA). Lipofectamine 2000 (Invitrogen) was used to transfect the above RNAs and plasmid into T-47D-CSCs.

### Cell viability assay

Cells were grown overnight in 96-well plates at a density of 5×10^3^ cells/well. Then, RNAs and plasmid vectors were transfected into the tumor cells. 24 h post transfection, cells were treated with cisplatin for 48 h. Subsequently, 20 μl 3-(4, 5-dimethylthiazol-2-yl)-2, 5-diphenyltetrazolium bromide (MTT) (5 mg/ml, Sigma-Aldrich, USA) was added to the medium and incubated at 37°C for 4 h. The supernatant was then removed, and 150 μL DMSO was added and thoroughly mixed. The absorbance at 570 nm was measured with a microplate reader (Sunrise Microplate Reader, TECAN, Switzerland).

### Luciferase reporter assay

To evaluate the function of miR-519d, the 3′ UTR of MCL-1 was amplified and inserted downstream of the luciferase reporter gene in the luciferase reporter pMIR-REPORT luciferase reporter vector (Ambion, Carlsbad, CA, USA). The mutant 3′UTR of MCL-1 was amplified using wild-type MCL-1 3 'UTR as the template, and the mutant plasmid was created by Site-Directed Mutagenesis Kit (TaKaRa). For luciferase reporter assays, the cells were co-transfected with miR-519d mimics and wild-type or mutant MCL-1 3′UTR, along with Renilla luciferase pRL-TK vector (Promega, USA) as an internal control. Following transfection for 48 h, the cells were collected and lysed using RIPA buffer (Cell Signaling Technology, USA). Luciferase activity was then measured by using Dual Luciferase Assay System (Promega) according to the manufacturer's instructions.

### Mitochondria isolation

For detection of cytochrome C and Smac/DIABLO, the mitochondria in cells were isolated using Mitochondria/Cytosol Fraction Kit (BioVision, USA) according to the manufacturer's guidance. The release of cytochrome C and Smac/DIABLO from mitochondria was measured using western blot analysis.

### Immunoprecipitation and western blot

Cells and tumor tissues were lysed using RIPA buffer, and then the lysates were incubated with antibody of Apaf-1 or Smac/DIABLO (Cell Signaling Technologies, USA) overnight at 4°C. Subsequently, the protein A agarose beads was added and incubated for 2 h. After washing the beads with cold RIPA buffer, proteins were removed from the beads by boiling in sodium dodecyl sulfate (SDS) sample buffer. For western blot analysis, the extracted proteins were separated by 12.5 % sodium dodecyl sulfate polyacrylamide gel electrophoresis (SDS-PAGE) and transferred to a PVDF membrane (Millipore, USA). The membranes were blocked with 5 % skim milk for 1 h at room temperature and then incubated overnight at 4°C with the primary antibodies (MCL-1, cleaved caspase-9, cleaved caspase-7, cleaved caspase-3, cytochrome C, second mitochondria-derived activator of caspase / direct IAP binding protein with low pI (Smac/DIABLO), apoptotic peptidase activating factor 1 (Apaf-1), X-linked inhibitor of apoptosis (Xiap) and β-actin, all of them purchased from Cell Signaling Technology). Subsequently, the membranes were incubated with a horseradish peroxidase (HRP)-conjugated secondary antibody (Cell Signaling Technology) for 2 h at room temperature. Signals were detected using enhanced chemiluminescence reagents (Thermo, USA).

### Detection of apoptosis and mitochondrial membrane potential (MMP, ΔΨ_m_)

Cell apoptosis measurement was measured by PI and Annexin-V staining using the FITC-Annexin V reagents according to the manufacturer's instructions (Sigma-Aldrich, USA). Mitochondrial membrane potential (MMP, ΔΨ_m_) was detected using 5,5′,6,6′-Tetrachloro-1,1′,3,3′-tetraethyl imidacarbo cyanine iodide (JC-1, Molecular Probes, USA) as an indicator [37] according to the manufacturer's instructions. Both the apoptosis and ΔΨ_m_ were analyzed by flow cytometry analysis (Becton Dickinson, USA).

### Xenografts

To conduct the stable T-47D cells which overexpress miR-519d, we purchased the recombinant lentivirus which contains miR-519d precusor sequence and empty viral vector (EV) from the Shanghai Genechem Co., Ltd. (Shanghai, China). The precusor sequence of miR-519d is as follows: 5′- UCCCAUGCUGUGACCCUCCAAAGGGAAGCGCUUUCUGUUUGUUUUC UCUUAAACAAAGUGCCUCCCUUUAGAGUGUUACCGUUUGGGA-3′. Routine T-47D cells were transfected with 5×10^5^ transducing units of lentivirus and then selected with 1 μg/ml puromycin for 2 weeks. The lentivirus-transfected T-47D cells were collected for the use of animal experiments. Female BALB/c nude mice (SPF, 4-5-week-old) were obtained from Shanghai Super-B&K Laboratory Animal Corp., Ltd. (Shanghai, China). Forty mice were divided into four groups (5 mice/group). For xenograft, two groups of mice were subcutaneously injected with 5×10^6^ T-47D cells transfected with empty viral vector with/without cisplatin treatment (EV+cisplatin or EV group, respectively). The other two groups of mice were subcutaneously injected with 5×10^6^ T-47D cells transfected with lentivirus carrying miR-519d with/without cisplatin treatment (miR-519d+cisplatin or miR-519d group, respectively). Cisplatin was administrated by intraperitoneal injection twice a week (3 mg/kg) after xenografts reached 0.5 cm in diameter. Tumor-bearing mice were euthanized on 31 days post-injection. Tumor volume (V) was calculated based on the equation of 1/2 × length × width^2^. The animal care and experimental protocols were approved by the Animal Care Committee of Xinxiang Medical University.

### Statistical analysis

All data are expressed as the mean ± standard deviation and carried out by three independent experiments. Statistical analysis was performed by Student's t-test using SPSS 13.0 software. Values of *P*<0.05 were considered significant.
